# A Rare Case of Eosinophilic Gastroenteritis and Ampullitis Leading to Benign Papillary Stenosis

**DOI:** 10.1155/2021/5597578

**Published:** 2021-03-09

**Authors:** Akwe Nyabera, Keya Shah, Nso Nso, Saphwat Eskaros, Negar Niknam

**Affiliations:** ^1^Icahn School of Medicine at Mount Sinai (NYC Health+Hospitals/Queens) Program, New York City, NY, USA; ^2^NYIT College of Osteopathic Medicine, Old Westbury, NY, USA

## Abstract

Eosinophilic gastroenteritis is characterized by eosinophilic infiltration of the gastrointestinal wall. There have been limited studies of eosinophilic infiltration involving the ampulla. We present a 70-year-old woman with a history of asthma, eosinophilic esophagitis, and eosinophilic sinusitis, who underwent work up for postprandial abdominal pain and abnormal liver function tests. The patient had various imaging studies done, including computed tomography (CT) scan, magnetic resonance imaging (MRI), and magnetic resonance cholangiopancreatography (MRCP). Dilated extrahepatic bile duct with distal tapering towards the ampulla was noted on MRCP and afterwards on endoscopic ultrasound (EUS). Endoscopic retrograde cholangiopancreatography (ERCP) revealed an inflamed major ampulla with benign papillary stenosis. The patient was treated with sphincterotomy, sphincteroplasty/dilation of distal common bile duct, and placement of an 11.5 Fr × 7 cm plastic stent into the bile duct. Additionally, she was started on prednisone, which was gradually tapered down. The patient demonstrated significant improvement with systemic steroid therapy. Liver function tests (LFTs) completely normalized after ERCP. Ampullitis leading to papillary stenosis secondary to eosinophilic infiltration of the major papilla is a rare manifestation of eosinophilic gastrointestinal disorders (EGIDs). Early diagnosis would lead to appropriate medical and endoscopic management.

## 1. Introduction

Eosinophilic gastroenteritis is characterized by eosinophilic infiltration of the gastrointestinal wall [1, 2]. The stomach and small intestine are the most commonly affected organs [3, 4]. There have been limited studies of eosinophilic infiltration involving the ampulla. We present a patient with abnormal liver function tests and abdominal pain who was found to have benign papillary stenosis secondary to eosinophilic infiltration of the major papilla. The clinical presentation of eosinophilic gastroenteritis varies widely. It may present similarly to irritable bowel syndrome, dyspepsia, pancreatitis, or appendicitis [3, 5]. Eosinophils are inhabitants of the gastrointestinal mucosal lining delivering a natural defense in cases of parasitic infections [2]. High cytotoxic granular proteins will release after excessive eosinophils and accumulate in the mucosal tissue causing serious tissue destruction [5]. Biopsy is an important tool for establishing a diagnosis [2, 6]. Multiple biopsies may be required due to the variable nature of the disease [2]. Eosinophilic gastroenteritis is a substantially treatable disease with the elimination of empirical food diets. A single course of steroid therapy is first line therapy; in cases of relapsing or refractory conditions, steroid-sparing immunosuppressive agents are recommended in association with biological agents [3, 5]. Treatment is largely dependent on the severity of symptoms and extent of disease [7].

## 2. Case Presentation

**Figure 1:**
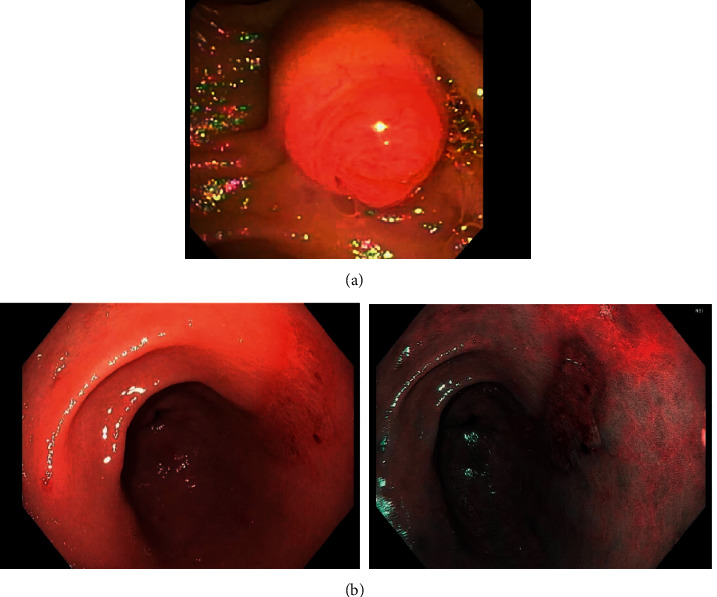
(a) Prominent and bulging major ampulla and (b) antral gastritis (superficial gastric ulcers).

A 70-year-old woman with a history of asthma, eosinophilic esophagitis, and eosinophilic sinusitis underwent work-up for postprandial abdominal pain, bloating, and dyspepsia. Her symptoms had been ongoing intermittently for 2 years. Laboratory results were significant for elevated eosinophils (19.4%) lipase (376 U/L), ALT (247 U/L), AST (332 U/L), and alkaline phosphatase (131 U/L). Initial EGD showed a hypertrophic major papilla, antral gastritis, and superficial gastric ulcers (Figures 1(a) and 1(b)).

**Figure 2:**
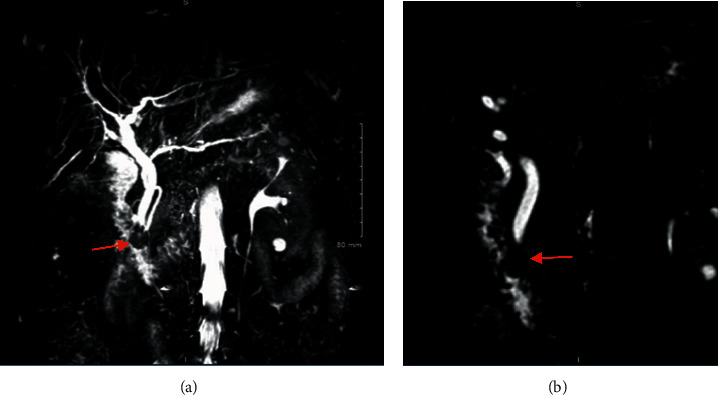
MRI/MRCP showing CBD dilatation without choledocholithiasis as well as papillary stenosis.

She underwent MRI/MRCP which revealed CBD dilatation without choledocholithiasis but with tapering of the distal CBD (Figure 2).

The common hepatic duct was dilated to 10 mm, and the CBD was dilated to 8 mm with tapering towards the ampulla. ERCP revealed an inflamed major ampulla with benign papillary stenosis.

The patient was treated with sphincterotomy, and placement of an 11.5 Fr × 7 cm plastic stent into the bile duct was done. After sphincterotomy, the sphincter tissue was thick and fibrotic. Biopsies were significant for squamous esophageal mucosa with mild chronic esophagitis and gastric mucosa with increased eosinophils (reaching 26/high power field). Ampullary tissue showed chronic inflammation with focal increased eosinophils in the lamina propria; however, the exact eosinophilic count in the ampulla was not reported. Repeat ERCP was performed with stent removal, as well as sphincteroplasty/dilation of the papilla and distal CBD resulting in resolution of the stenosis. Repeat biopsies of the stomach and the ampulla showed benign glandular tissue with no eosinophils. Colonoscopy had been performed 5 years prior to presentation; findings were benign and did not show any eosinophilic infiltration of the colon.

Additionally, she was started on prednisone 40 mg daily, which was gradually tapered down by 10 mg a week. The patient demonstrated significant improvement with systemic steroid therapy. Liver function tests completely normalized, and eosinophil count trended down, but remained persistently elevated (13%). She was kept on 40 mg of pantoprazole twice a day and 2 mg of budesonide daily. Patient was monitored over six months, LFTs remained normal, and symptoms were controlled.

## 3. Discussion

We present a rare case of eosinophilic infiltration of the major ampulla which resulted in benign papillary stenosis. EGIDs represent a spectrum of inflammatory conditions classified by the infiltration of eosinophils in the gastrointestinal tract that manifests with variable clinical features [8]. Eosinophilic infiltration can involve any part of the gastrointestinal tract from the mouth to the anus and can infiltrate the gastrointestinal wall from the mucosa to the serosa [6]. Prevalence of EGIDs shows a primarily female predominance. Overall, EGIDs are rare, with less than 50,000 people affected in the US [9]. Manifestations are dependent on the location and extent of gastrointestinal involvement and more commonly present with abdominal pain, nausea, early satiety, vomiting, diarrhea, and weight loss [10]. Extraintestinal manifestations can be present in many patients such as seasonal allergies, atopy, and eczema [8].

Several imaging modalities and endoscopic procedures such as CT, MRI/MRCP, EGD, and EUS can be utilized to provide further insight into the condition when it involves the pancreatobiliary system [2]. In our patient, magnetic MRI/MRCP and EUS revealed CBD dilatation without choledocholithiasis but tapering of distal CBD towards the ampulla. EGD revealed antral gastritis, superficial gastric ulcers, and prominent major ampulla. ERCP revealed an inflamed major ampulla and benign papillary stenosis.

Biopsy is an important tool for establishing a diagnosis [2, 6]. Diagnosis of EGIDs often involves the presence of eosinophilic infiltration of the gastrointestinal tract on multiple biopsies as well as the absence of another disease process causing intestinal eosinophilia [2]. Similar to the patient's findings, gross endoscopic findings are generally nonspecific and may reveal mucosal edema, patchy erythematous changes, and superficial ulcerations, often mistaken for other gastrointestinal disorders [7]. The pathogenesis of EGIDs is not well understood and is thought to be related to the interaction between chemokines and eosinophils causing the release of cytokines such as interleukin-3, interleukin-5, and granulocyte colony-stimulating factor [11]. High cytotoxic granular proteins will release after excessive eosinophils accumulate in the mucosal tissue causing serious tissue destruction [5].

Treatment may vary depending on organ involvement, severity of symptoms, and extent of the disease [7]. In our patient, secondary to papillary stenosis, treatment was targeted to the biliary tree. The patient underwent endoscopic therapy with sphincterotomy, sphincteroplasty, and stent placement, as well as administration of systemic steroids. Systemic steroid therapy usually improves the symptoms of eosinophilic gastroenteritis, but in our patient, the ampulla was fibrotic appearing (likely secondary to sequalae of chronic inflammation), and the benign papillary stenosis was unlikely to resolve with steroid therapy alone. It required additional sphincterotomy, stent placement, and sphincteroplasty (balloon dilation of the papillae and the distal CBD). The same mechanism is also observed in esophageal stenosis in patients with eosinophilic esophagitis. A fibrotic stenosis of esophagus in the setting of eosinophilic esophagitis would require dilation as well as medical treatment with steroids and proton pump inhibitor [12].

Other treatment options include modified diets due to food sensitivities, glucocorticoids, and azathioprine to inhibit inflammatory eosinophilic growth factors, leukotriene receptor antagonists to diminish eosinophil chemoattraction, and biologic agents against targeted inflammatory mediators [7]. Lirentelimab, a newer agent, is an anti-Siglec-8 antibody that depletes eosinophils and inhibits mast cells. It has been used in phase 2 clinical trials with favorable outcomes. However, further studies are needed to validate these results [13].

Ampullitis leading to papillary stenosis secondary to eosinophilic infiltration in the major papilla is a rare manifestation of EGIDs. Early diagnosis would lead to appropriate medical and endoscopic management and perhaps prevention of sever strictures.
